# Supplementary material for: Signatures of a surface spin–orbital chiral metal
Source: Nature. 2024 Feb 7;626(8000):752–8. doi: 10.1038/s41586-024-07033-8 (PMC10881390; doi:10.1038/s41586-024-07033-8)
Supplement: Supplementary file 1 — Supplementary Sections 1–3, including Supplementary Figs. 1–5 and References. Section 1 contains details regarding the model and broken symmetry phases with chiral currents. Section 2 contains details regarding spin-resolved circularly polarized ARPES matrix elements and orbital angular momentum. Section 3 contains details regarding the current driven phase by Coulomb interaction. [file 41586_2024_7033_MOESM1_ESM.pdf]

nature portfolio

https://doi.org/10.1038/s41586-024-07033-8

Supplementary information

Signatures of a surface spin–orbital chiral metal

In the format provided by the authors and unedited

Supplementary Information: Signatures of a surface spin-orbital chiral metal

Federico Mazzola**,[1,2,*] Wojciech Brzezicki**,[3,4,†] Maria Teresa Mercaldo,[5] Anita Guarino,[6] Chiara Bigi,[7] Jill A. Miwa,[8] Domenico De Fazio,[1] Alberto Crepaldi,[9] Jun Fujii,[2] Giorgio Rossi,[10,2] Pasquale Orgiani,[2] Sandeep Kumar Chaluvadi,[2] Shyni Punathum Chalil,[2] Giancarlo Panaccione,[2] Anupam Jana,[2] Vincent Polewczyk,[2] Ivana Vobornik,[2] Changyoung Kim,[11] Fabio Miletto Granozio,[12,13] Rosalba Fittipaldi,[6] Carmine Ortix,[5] Mario Cuoco,[6,‡] and Antonio Vecchione[6,§]

[1]*Department of Molecular Sciences and Nanosystems, Ca' Foscari University of Venice, 30172 Venice, Italy*

[2]*Istituto Officina dei Materiali, Consiglio Nazionale delle Ricerche, Trieste I-34149, Italy*

[3]*Institute of Theoretical Physics, Jagiellonian University, ulic, S. Łojasiewicza 11, PL-30348 Kraków, Poland*

[4]*International Research Centre MagTop, Institute of Physics, Polish Academy of Sciences, Aleja Lotników 32/46, PL-02668 Warsaw, Poland*

[5]*Dipartimento di Fisica "E. R. Caianiello", Università di Salerno, IT-84084 Fisciano (SA), Italy*

[6]*CNR-SPIN, c/o Universitá di Salerno, IT-84084 Fisciano (SA), Italy*

[7]*Synchrotron SOLEIL, F-91190 Saint-Aubin, France*

[8]*Department of Physics and Astronomy, Interdisciplinary Nanoscience Center, Aarhus University, 8000 Aarhus C, Denmark*

[9]*Dipartimento di Fisica, Politecnico di Milano, Piazza Leonardo Da Vinci 32, Milano 20133, Italy*

[10]*Dipartimento di Fisica, Università degli studi di Milano, IT-20133 Milano, Italy*

[11]*Department of Physics and Astronomy, Seoul National University, Seoul, 08826, Korea*

[12]*CNR-SPIN, I-80126 Napoli, Italy*

[13]*Dipartimento di Fisica, Università di Napoli, Napoli, Italy*

I. MODEL AND BROKEN SYMMETRY PHASES WITH CHIRAL CURRENTS

We consider a two-dimensional system whose electronic states at the Fermi level are described by d-orbitals belonging to the t_{2g} manifold (d_{xy},d_{xz},d_{yz}) and can accommodate electronic phases which are marked by non-trivial loop currents.

The model Hamiltonian for the surface of the Sr_2RuO_4 is compatible with the C_{4v} point group symmetry. For the t_{2g} manifold of the d_a orbitals we introduce the components of the local orbital angular momentum. For the spin space, hereafter, we employ the Pauli matrices $\hat{s}_{j=x,y,z}$. Assuming that the basis of the local creation operator of electrons for the d-orbitals for a given spin orientation is $\hat{d}^{\dagger}_{\boldsymbol{k}} = [c^{\dagger}_{xy,\boldsymbol{k}}, c^{\dagger}_{yz,\boldsymbol{k}}, c^{\dagger}_{xz,\boldsymbol{k}}]$, we have that the components of the orbital angular momentum are given by:

$$\hat{L}_x = \begin{pmatrix} 0 & -i & 0 \\ i & 0 & 0 \\ 0 & 0 & 0 \end{pmatrix} \qquad \hat{L}_y = \begin{pmatrix} 0 & 0 & -i \\ 0 & 0 & 0 \\ i & 0 & 0 \end{pmatrix} \qquad \hat{L}_z = \begin{pmatrix} 0 & 0 & 0 \\ 0 & 0 & -i \\ 0 & i & 0\,. \end{pmatrix} \tag{1}$$

with $\hat{L}_i$ fulfilling the usual angular momentum algebra.

Then, in the complete spin-orbital basis, given by $\hat{C}^{\dagger}_{\boldsymbol{k}} = [c^{\dagger}_{xy,\uparrow\boldsymbol{k}}, c^{\dagger}_{yz,\uparrow\boldsymbol{k}}, c^{\dagger}_{zx,\uparrow\boldsymbol{k}}, c^{\dagger}_{xy,\downarrow\boldsymbol{k}}, c^{\dagger}_{yz,\downarrow\boldsymbol{k}}, c^{\dagger}_{zx,\downarrow\boldsymbol{k}}]$, the Hamiltonian can be generally expressed as

$$\hat{\mathcal{H}} = \sum_{\boldsymbol{k}} \hat{C}^{\dagger}_{\boldsymbol{k}} \hat{H}(\boldsymbol{k}) \hat{C}_{\boldsymbol{k}}, \tag{2}$$

with $\hat{H}(\mathbf{k})$ given by

$$\begin{aligned} \hat{H} = \sum_{\boldsymbol{k}} [(\epsilon_{xy}(k_x, k_y) - \mu)\mathbb{P}_z + (\epsilon_{xz}(k_x, k_y) - \mu)\mathbb{P}_y + (\epsilon_{yz}(k_x, k_y) - \mu)\mathbb{P}_x + \\ + \epsilon_{xz,yz}(k_x, k_y)(\hat{L}_x\hat{L}_y + \hat{L}_y\hat{L}_x) + \alpha_{OR}(\sin(k_x)\,\hat{L}_y - \sin(k_y)\hat{L}_x)]\hat{\sigma}_0 + \lambda_{\mathrm{SO}}\hat{\boldsymbol{L}} \cdot \hat{\boldsymbol{\sigma}}\,. \end{aligned} \tag{3}$$

We recall that $\mathbb{P}_a = (\hat{L}^2 - 2\hat{L}_a^2)/2$ is the projector on the orbital state with a given distribution that is perpendicular to the a direction (e.g. for $a = z$ the projection is on the xy orbital configuration). Here, μ is the chemical potential. The term proportional to $\epsilon_{xz,yz}(k_x, k_y)$ corresponds

* federico.mazzola@unive.it

† w.brzezicki@uj.edu.pl

‡ mario.cuoco@spin.cnr.it

§ antonio.vecchione@spin.cnr.it

to the symmetry allowed hybridization processes of the (xz, yz) orbitals along the $[110]$ and $[\bar{1}10]$ orientations. The choice of the following electronic parameters reproduces with good accuracy the experimental profile of the Sr_2RuO_4 Fermi lines at the surface as measured by ARPES (as in this paper and in Ref. 1, see e.g. Fig. 2):

$$\begin{aligned}
\epsilon_{xy}(k_x, k_y) =& -2t_3[\cos(k_x) + \cos(k_y)] - 4t_4 \cos(k_x)\cos(k_y) - 2t_5[\cos(2k_x) + \cos(2k_y)] \\
\epsilon_{yz}(k_x, k_y) =& -2t_2 \cos(k_x) - 2t_1 \cos(k_y) \\
\epsilon_{xz}(k_x, k_y) =& -2t_1 \cos(k_x) - 2t_2 \cos(k_y) \\
\epsilon_{xz,yz}(k_x, k_y) =& -4t_6 \sin(k_x)\sin(k_y)
\end{aligned}$$

with $t_1 = 0.145$, $t_2 = 0.016$, $t_3 = 0.08$, $t_4 = 0.039$, $t_5 = 0.005$, $t_6 = 0.001$, $\mu = 0.122$, $\lambda_{\mathrm{SO}} = 0.035$, in units of eV [1]. We assume a weak amplitude for the inversion symmetry breaking term $\alpha_{OR} \sim 0.01$ eV, since the induced splitting is below the experimental resolution. The term proportional to α_{OR} represents the orbital Rashba interaction [2–5] and couples the atomic angular momentum $\mathbf{L}$ with the crystal wave-vector $\mathbf{k}$ due to the breaking of inversion symmetry at the surface.

Then, in order to take into account the breaking of time-reversal and mirror symmetry, we consider the spin-orbital chiral current phase along the $l = [110]$ direction, as given the following term in the Hamiltonian:

$$\hat{j}_{\mathrm{so}} = \boldsymbol{g}_{\mathrm{so}} \cdot (\hat{\boldsymbol{L}} \times \hat{\boldsymbol{\sigma}}) \sin k_l \,. \tag{4}$$

Here, we take a representative $\boldsymbol{g}_{\mathrm{so}}$ vector with amplitude $|\boldsymbol{g}_{\mathrm{so}}| = 0.04$ eV, with generic coefficients, so that it does not point in any high-symmetry direction in the lattice. The outcome of the orbital and spin-orbital quadrupole moments within the Brillouin zone is not qualitatively affected by the choice of the coefficients. This current term can arise by a mean-field decoupling of the Coulomb interaction for nearest-neighbor Ru centers (see Sect. IV). In Fig. 1 we report the orbital and spin-orbital textures for all the bands assuming different types of broken symmetry chiral phases marked by orbital and spin-orbital quadrupole currents.

In order to capture the different behavior of the orbital and spin-orbital moments in the presence of chiral currents one can consider the equation of motion associated with the expectation values of the spin and orbital moments for a given eigenstate at momentum k. In general, the time dependent evolution of the spin and orbital angular momentum is provided by the equation of motion $\frac{dM}{dt} = \boldsymbol{\tau_M}$ with $\boldsymbol{M} = \boldsymbol{s}, \boldsymbol{L}$ and $\boldsymbol{\tau}_M$ being the corresponding torques. For a given eigenstate

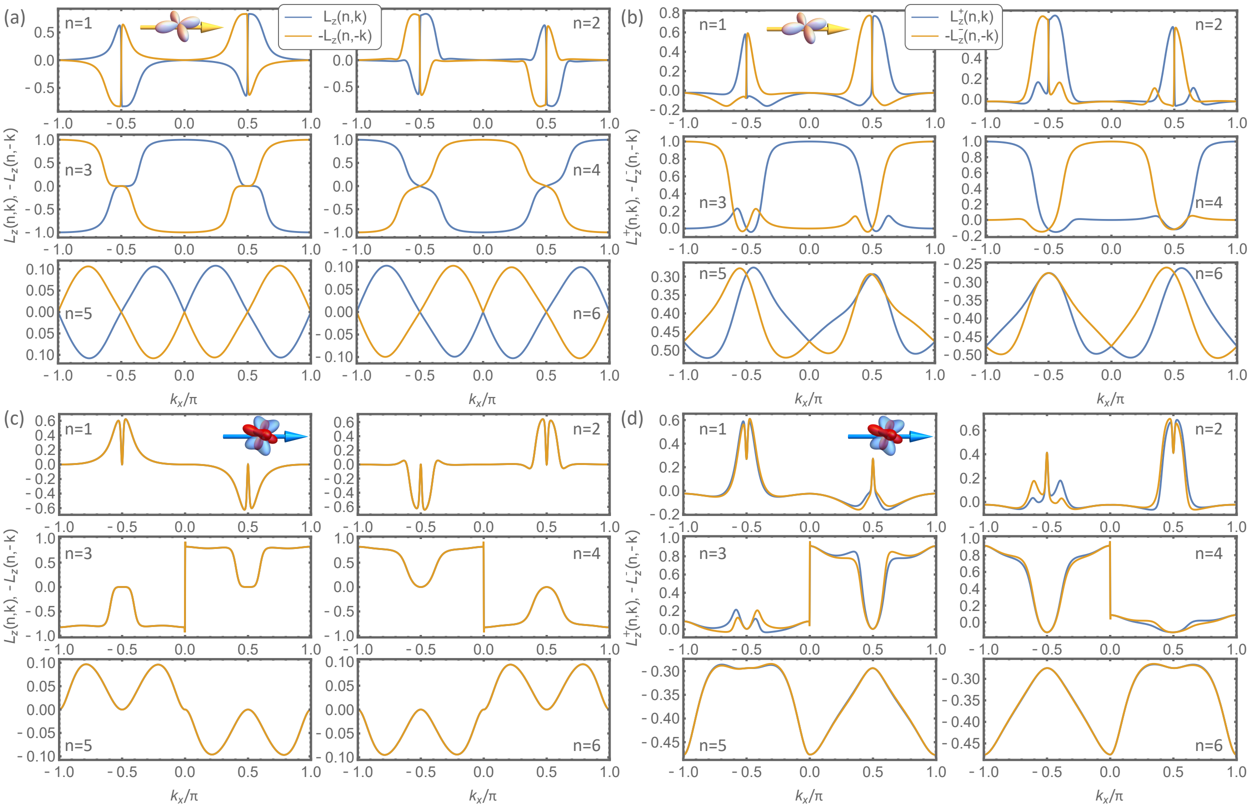

FIG. 1. **Orbital and spin-orbital textures in the presence of chiral currents.** **a** Electronic phase with chiral orbital-quadrupole currents: amplitude of the orbital angular momentum $L_z(n,k)$ of all bands $|\psi_{n,k}\rangle$ evaluated along the $\Gamma - X$ direction ($L_z(n,k) = \langle\psi_{n,k}|\hat{L}_z|\psi_{n,k}\rangle$). For clarity we plot both $L_z(n,k)$ and $-L_z(n,-k)$ for any given momentum k to directly compare the amplitudes at opposite momenta. **b** Electronic phase with chiral orbital-quadrupole currents: amplitude of the spin projected orbital angular momentum ($L_z^{\pm}(n,k) = \langle\psi_{n,k}|(1 \pm \hat{s}_z)\hat{L}_z|\psi_{n,k}\rangle$). The amplitudes of $L_z(n,k)$ and $L_z^{\pm}(n,k)$, displayed in **a** and **b**, do not show any symmetry and do not match at k and $-k$. **c** Electronic phase with chiral spin-orbital quadrupole currents with antisymmetric $L\,S$ combination: $L_z(n,k)$ and $L_z(n,-k)$ coincide. **d** Electronic phase with chiral spin-orbital quadrupole currents with antisymmetric $L\,S$ combination: spin projected orbital moment $L_z^{\pm}(n,k)$ at opposite momenta are unequal in amplitude.

$|\psi_{nk}\rangle$ of the band n at momentum k, one can write a torque equation for the amplitudes of the spin $\boldsymbol{s}_{nk} = \langle\psi_{nk}|\hat{\boldsymbol{\sigma}}|\psi_{nk}\rangle$ and orbital $\boldsymbol{L}_{nk} = \langle\psi_{nk}|\hat{\boldsymbol{L}}|\psi_{nk}\rangle$ moments. The equation of motion reads as $\frac{d\boldsymbol{M}_{nk}}{dt} = \boldsymbol{\tau}_{nk}^{M}$ with $\mathbf{M} = \mathbf{s},\mathbf{L}$ and $\boldsymbol{\tau}_{nk}^{M} = i\langle\psi_{nk}|[H,\hat{\boldsymbol{M}}]|\psi_{nk}\rangle$. Now, since we deal with equilibrium configurations in time-independent conditions the amplitudes of the spin and orbital moments have to fulfill the relations $\tau_{nk}^{M} = 0$ with $M = s, L$. These relations in turn set out the amplitude of the

spin and orbital moments.

For convenience and clarity, we introduce a Cartesian reference xyz and, without loss of generality, we assume that the chiral current in the broken symmetry phase flows parallel to the x-direction. The spin-orbital current in Eq. (4) can be then generally expressed as

$$\hat{j}_{\text{so}} = \left[g_{so}^{x}(\hat{L}_y\hat{\sigma}_z - \hat{L}_z\hat{\sigma}_y) + g_{so}^{y}(\hat{L}_z\hat{\sigma}_x - \hat{L}_x\hat{\sigma}_z) + g_{so}^{z}(\hat{L}_x\hat{\sigma}_y - \hat{L}_y\hat{\sigma}_x)\right] \sin k_x \tag{5}$$

Furthermore, it is also convenient to write the Hamiltonian in the following compact form:

$$\hat{H} = H_0 + \hat{H}_{so} + \hat{j}_{\text{so}} \tag{6}$$

with H_0 containing the kinetic and the orbital Rashba coupling terms and $\hat{H}_{so} = \lambda_{so}\hat{\boldsymbol{L}} \cdot \hat{\boldsymbol{\sigma}}$. We notice that H_0 preserves the vertical mirrors (M_x and M_y) and the time-reversal symmetries. Then, we point out that among the components of $\hat{j}_{\text{so}}$, the term related to g_{so}^{x} is the source of chiral symmetry breaking, as all the mirror symmetries are broken. This is because the components of $\hat{\mathbf{L}}$ and $\hat{\boldsymbol{\sigma}}$ are pseudovectors and are perpendicular to the current flow direction. Instead, the other components, related to g_{so}^{y} and g_{so}^{z}, preserve the M_x vertical mirror symmetry.

Let us now consider the spin and orbital amplitudes of the torque by taking for instance the corresponding orientations along the x-direction. For this configuration, the commutators of the Hamiltonian with the x component of spin and orbital angular momentum yield

$$[\hat{H}_0, \hat{\sigma}_x] = 0 \tag{7}$$

$$\left[\hat{H}_{so}, \hat{\sigma}_x\right] = i\lambda_{so}(\hat{L}_z\hat{\sigma}_y - \hat{L}_y\hat{\sigma}_z) \equiv i\lambda_{so}\hat{A} \tag{8}$$

$$\left[\hat{j}_{so}, \hat{\sigma}_x\right] = i\left[h_x(k_x)\hat{B}_x + h_y(k_x)\hat{B}_y + h_z(k_x)\hat{B}_z)\right] \tag{9}$$

where $\hat{B}_x = \hat{L}_y\hat{\sigma}_y + \hat{L}_z\hat{\sigma}_z$, $\hat{B}_y = \hat{L}_x\hat{\sigma}_y$, $\hat{B}_z = -\hat{L}_x\hat{\sigma}_z$ and $h_i(k_x) = g_{so}^{i}\sin(k_x)$ with $i = x, y, z$. On the basis of these relations, the equilibrium condition for the spin torque yields:

$$\tau_{nk}^{s_x} = \tau_{nk}^{s_x,so} + \sum_{i=x,y,z} \tau_{nk}^{s_x,j_{so}^{i}} = 0 \tag{10}$$

with

$$\tau_{nk}^{s_x,so} = -\lambda_{so}\langle\psi_{nk}|\hat{A}|\psi_{nk}\rangle \tag{11}$$

$$\tau_{nk}^{s_x,j_{so}^{i}} = -h_i(k_x)\langle\psi_{nk}|\hat{B}_i|\psi_{nk}\rangle \,. \tag{12}$$

We notice that in the equation for $\tau_{nk}^{s_x}$ the torque arising from the x component of the spin-orbital current (i.e. $\tau_{nk}^{s_x,j_{so}^{i}}$) breaks the M_x mirror symmetry. Instead, the terms related to $g_{so}^{y,z}$ are mirror-symmetric. We also observe that in the absence of the spin-orbital current the spin torque due to

the spin-orbit coupling is identically zero. This implies that the spin and orbital moments must be collinear at any k. On the other hand, the presence of the chiral spin-orbital currents leads to a deviation from the collinearity between s and L.

Let us now consider the analogous equations for the orbital moment. For the commutators with $\hat{L}_x$ we have:

$$[\hat{H}_0, \hat{L}_x] = -if_y(\mathbf{k})\hat{L}_z - i\sum_{a=x,y,z}(g_{ay}\{\hat{L}_a, \hat{L}_z\} - g_{zy}\{\hat{L}_a, \hat{L}_y\} \;\equiv\; i\hat{Q}(\mathbf{k}) \tag{13}$$

$$\left[\hat{H}_{so}, \hat{L}_x\right] = -i\lambda_{so}\hat{A} \tag{14}$$

$$\left[\hat{j}_{so}, \hat{L}_x\right] = i(-h_x(k_x)\hat{B}_x + h_y(k_x)\hat{C}_y + h_z(k_x)\hat{C}_z) \tag{15}$$

with $\hat{C}_y = \hat{L}_y\hat{\sigma}_x$ and $\hat{C}_z = \hat{L}_z\hat{\sigma}_x$. Here, the coefficient $f_y(\mathbf{k}) = \alpha_{OR}\sin(k_x)$ is related to the orbital Rashba coupling. Hence, the equation for the orbital moment torque becomes:

$$\tau_{nk}^{L_x} = \tau^{L_x,0} + \tau_{nk}^{L_x,so} + \sum_{i=x,y,z}\tau_{nk}^{L_x,j^i_{so}} = 0 \tag{16}$$

with

$$\tau_{nk}^{L_x,0} = -\langle\psi_{nk}|\hat{Q}(\mathbf{k})|\psi_{nk}\rangle \tag{17}$$

$$\tau_{nk}^{L_x,so} = \lambda_{so}\langle\psi_{nk}|\hat{A}|\psi_{nk}\rangle \tag{18}$$

$$\tau_{nk}^{L_x,j^x_{so}} = h_x(k_x)\langle\psi_{nk}|\hat{B}_x|\psi_{nk}\rangle \tag{19}$$

$$\tau_{nk}^{L_x,j^{i=y,z}_{so}} = -h_i(k_x)\langle\psi_{nk}|\hat{C}_i|\psi_{nk}\rangle\,. \tag{20}$$

Now, it is interesting to observe that $\tau_{nk}^{s_x,so} = -\tau_{nk}^{L_x,so}$ and $\tau_{nk}^{s_x,j^i_{so}} = -\tau_{nk}^{L_x,j^i_{so}}$. This relation is a hallmark of the phase with spin-orbital quadrupole currents. Taking into account these relations and the Eq. (10) for the spin torque, one can replace $\tau_{nk}^{s_x,so}$ and $\tau_{nk}^{s_x,j^i_{so}}$ in the Eq. (16) with the combination of $\tau_{nk}^{s_x,j^{a=y,z}_{so}}$. Hence, one can deduce an equation for the orbital torque that contains only terms that are mirror symmetry compatible. This implies that the expectation value of the orbital moment has a definite parity as for mirror-symmetric systems.

Let us now consider the case of the orbital quadrupole current:

$$\hat{j}_o = \left[g_o^x(\hat{L}_y\hat{L}_z + \hat{L}_z\hat{L}_y) + g_o^y(\hat{L}_z\hat{L}_x + \hat{L}_x\hat{L}_z) + g_o^z(\hat{L}_x\hat{L}_y + \hat{L}_y\hat{L}_x)\right]\sin k_x\,. \tag{21}$$

As for the case of the spin-orbital quadrupole current, the term related to g_o^x break both the horizontal (M_z) and vertical (M_y and M_x) mirror symmetries, while for the other current components, we have that g_o^y and g_o^z preserve M_x. For this configuration one can deduce that the spin torque

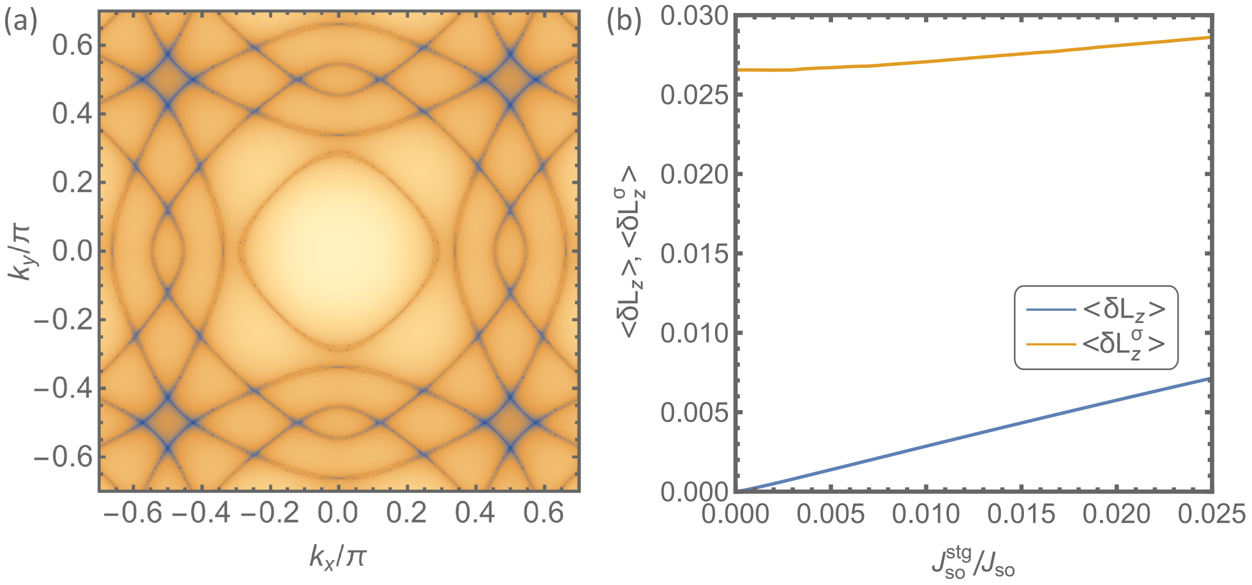

FIG. 2. **Fermi surface in the presence of the staggered octahedral rotation and average asymmetry of orbital and spin-projected orbital moment.** **a** Fermi surface. **b** k-integrated and band-averaged orbital moment $\langle \delta L_z \rangle = \frac{1}{12} \sum_n \int |L_z(n,k) + L_z(n,-k)| dk$ and $\langle \delta L_z^\sigma \rangle = \frac{1}{12} \sum_n \int |L_z^+(n,k) + L_z^-(n,-k)| dk$ as a function of the ratio of the staggered spin-orbital quadrupole current j_{so}^{stg} with respect to the homogeneous spin-orbital quadrupole current j_{so} component.

is not affected (i.e. $\hat{\sigma}$ commutes with $\hat{j}_o$) and that the orbital part includes all the torques due to the $g_o^{a=x,y,z}$ current components. Then, the resulting orbital moment has to explicitly manifest a breaking of the mirror perpendicular to the current flow as demonstrated in Fig. 1.

A. Surface reconstruction

To take into account the surface reconstruction due to the staggered octahedral rotation we double the unit cell, in order to include both sublattices of the square lattice within the unit cell. Then, the term related to the octahedral rotation can be included in the Hamiltonian as a staggered potential for the d-states at the Ru site of the form:

$$\hat{V}_{\text{stg}} = \eta_{\text{stg}} \hat{\tau}_z \left(\hat{L}_x \hat{L}_y + \hat{L}_y \hat{L}_x \right), \tag{22}$$

where τ_z is a sublattice pseudo-spin operator given by the corresponding Pauli matrix and η_{stg} is a characteristic energy scale of the rotation pattern. This term leads to a staggered splitting of the (xz, yz) orbitals at the Ru site that arises due to the local vertical mirrors symmetry breaking

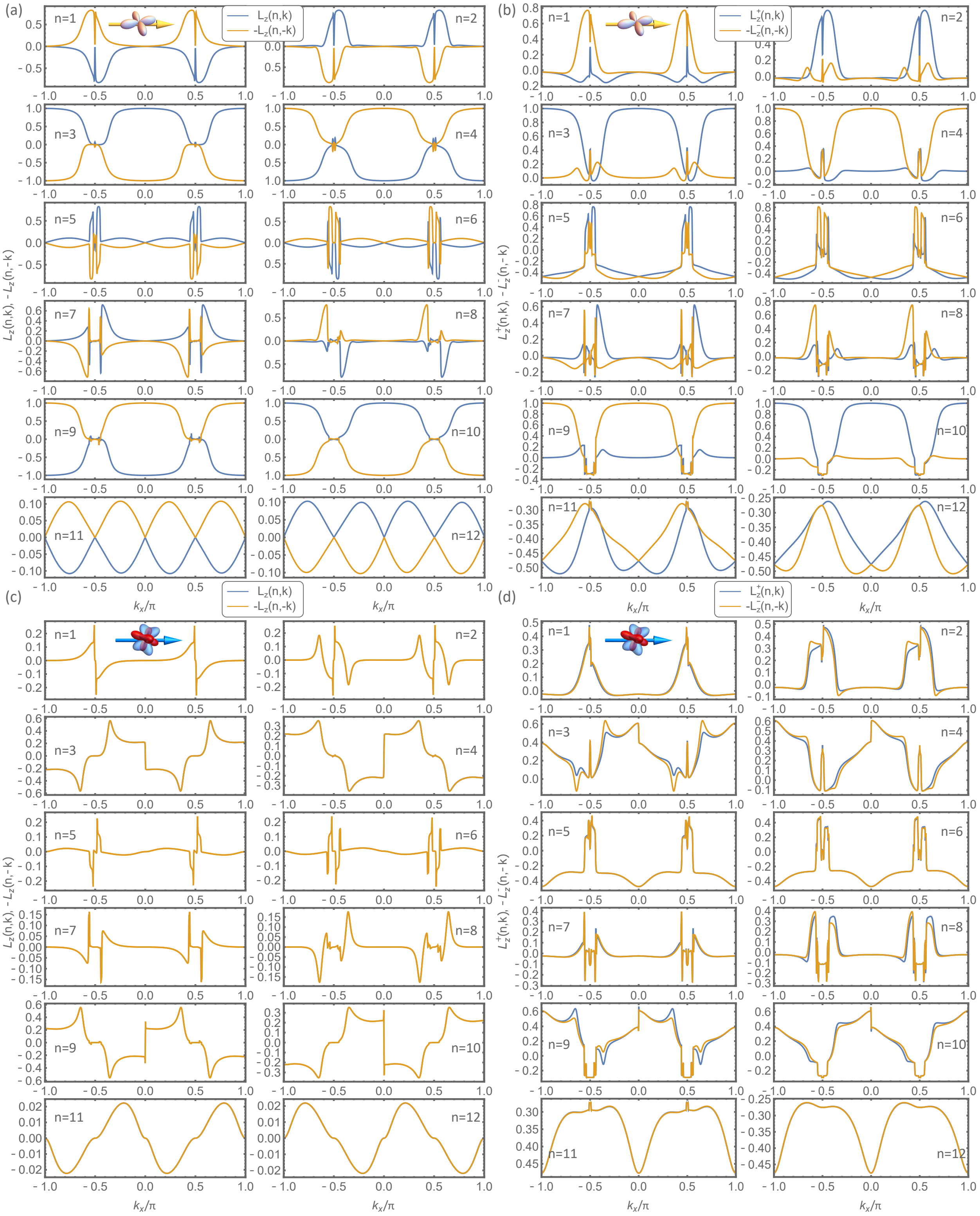

FIG. 3. **Orbital and spin-orbital moments in the presence of uniform chiral currents and octahedral rotation pattern.** Chiral orbital-quadrupole currents: **a** orbital $L_z(n,k)$ for all bands $|\psi_{n,k}\rangle$ evaluated along the $k_y = 0$ direction $(\Gamma - X)$, **b** spin-orbital $L_z^{\pm}(n,k)$. Chiral spin-orbital quadrupole currents: **c** orbital $L_z(n,k)$ of all bands $|\psi_{n,k}\rangle$ evaluated along the $k_y = 0$ direction, **d** spin-orbital $L_z^{\pm}(n,k)$.

resulting from the rotated octahedra. Here, for the computation of the electronic structure, we use a representative value for $\eta_{\text{stg}} = 5$ meV. Variation of the strength of η_{stg} does not alter the qualitative outcome of the analysis. We also introduce a staggered spin-orbital current component as given by the following contribution:

$$\hat{j}_{\text{so}}^{\text{stg}} = \hat{\tau}_z \left[\boldsymbol{g}_{\text{so}}^{\text{stg}} \cdot (\hat{\boldsymbol{L}} \times \hat{\boldsymbol{\sigma}}) \sin k_l \right] . \quad (23)$$

Hence, regarding the chiral current broken symmetry phase, we assume an amplitude modulation of the spin-orbital current along the [110] direction so that it is given by $j_{+}^{so} = j_{so} + j_{so}^{\text{stg}}$ when connecting Ru sites in octahedra that are clockwise rotated and $j_{-}^{so} = j_{so} - j_{so}^{\text{stg}}$ for anticlockwise rotated octahedra.

We have thus considered the possibility of having an amplitude modulation of the spin-orbital quadrupolar currents that follows the surface reconstruction due to the rotation of the RuO_6 octahedra. Then, it is expected that the ground state hosts chiral spin-orbital quadrupole currents which are not spatially homogeneous, being affected by the structural change of the octahedra, that result into different current amplitudes when linking ruthenium sites with inequivalent octahedral rotation. We have analyzed this configuration as a function of the current amplitude unbalance associated with the two sublattices. For such a current pattern we find that the band resolved dichroic asymmetry in the reconstructed electronic states (Fig. 2 a) is not exactly zero (Fig. 2 b) as found for the uniform current configuration. In order to get an overall estimate of the difference between the orbital and spin-projected orbital moments as a function of the current sublattice unbalance, we have followed their integrated values (i.e. summing up all the band contributions and integrating along the $\Gamma - X$ line). The outcome is reported in Fig. 2 b where one can clearly see that a non-vanishing orbital moment is induced by the spatially modulated chiral currents. Nevertheless, the asymmetry for the orbital moment turns out to be significantly smaller than that related to the spin projected one. For instance, for a spatial modulated configuration of the spin-orbital current with a sublattice unbalance of around 3% we find that the integrated value of the spin-projected orbital moment is about five times larger than that of the orbital moment. The point here is that the spatially homogeneous chiral spin-orbital current would lead to exact zero asymmetries in the dichroic amplitude due to the balance among the torques arising from the chiral current and that one arising from the spin-orbit coupling. Now, since the spin-orbit coupling at the Ru site is homogeneous, a spatial dependent amplitude of the chiral spin-orbital current cannot be

compensated and a non-vanishing amplitude of the dichroic signals can occur. Due to the surface reconstruction of the electronic states in the Sr_2RuO_4 one might argue that small deviations of the dichroic signals from zero can be consistent with the theoretical prediction when considering the orthorhombic configuration due to the staggered octahedral rotations around the c-axis. The main finding is that one can also account for an amplitude asymmetry of the dichroic signal keeping however a significantly larger spin-dichroic asymmetry as compared to the dichroic one.

Finally, we have verified that for a uniform configuration of the spin-orbital quadrupole currents the asymmetry of the orbital moment at k and $-k$ is identically zero irrespective of the surface reconstruction (Fig. 3).

B. C_4 rotational invariant spin-orbital chiral phases

We have considered states with spin-orbital chiral loop currents that preserve the C_4 rotational symmetry (see Fig. 4). These configurations lead to a symmetric dichroic amplitude and an asymmetric spin-dichroic value as related to the orbital moment $L_z(n,k)$ and spin projected orbital orbital moment $L_z^{\pm}(n,k)$ for all bands $|\psi_{n,k}\rangle$ evaluated along the $k_y = 0$ direction. In particular, due to the C_4 symmetries the orbital moment $L_z(n,k)$ can be vanishing. This implies that for this type of configurations we also expect a large difference between the asymmetry in the dichroic and spin-dichroic signals.

C. Canted antiferromagnetic order

We consider the case of a magnetic order with a canted antiferromagnetic configuration. The broken symmetry state is introduced by an effective magnetic term in the spin channel with uniform/staggered magnetization:

$$H_{\text{mag}} = g\mu_B \left(\hat{\tau}_0 \boldsymbol{M}_{\text{uni}} + \hat{\tau}_z \boldsymbol{M}_{\text{stg}}\right) \cdot \hat{\boldsymbol{\sigma}} \,, \tag{24}$$

with $\boldsymbol{M}_{\text{uni}}$ and $\boldsymbol{M}_{\text{stg}}$ being the uniform and staggered magnetization components. We have computed, for a representative antiferromagnetic configuration with canted moments, the orbital and spin-resolved orbital moment for all the bands crossing the Fermi level (Fig. 5). As one case see from the inspection of the results in Fig. 5 the orbital and spin-projected orbital moments exhibit a sizable asymmetry when comparing the amplitudes at k and $-k$. This is a general feature of all the

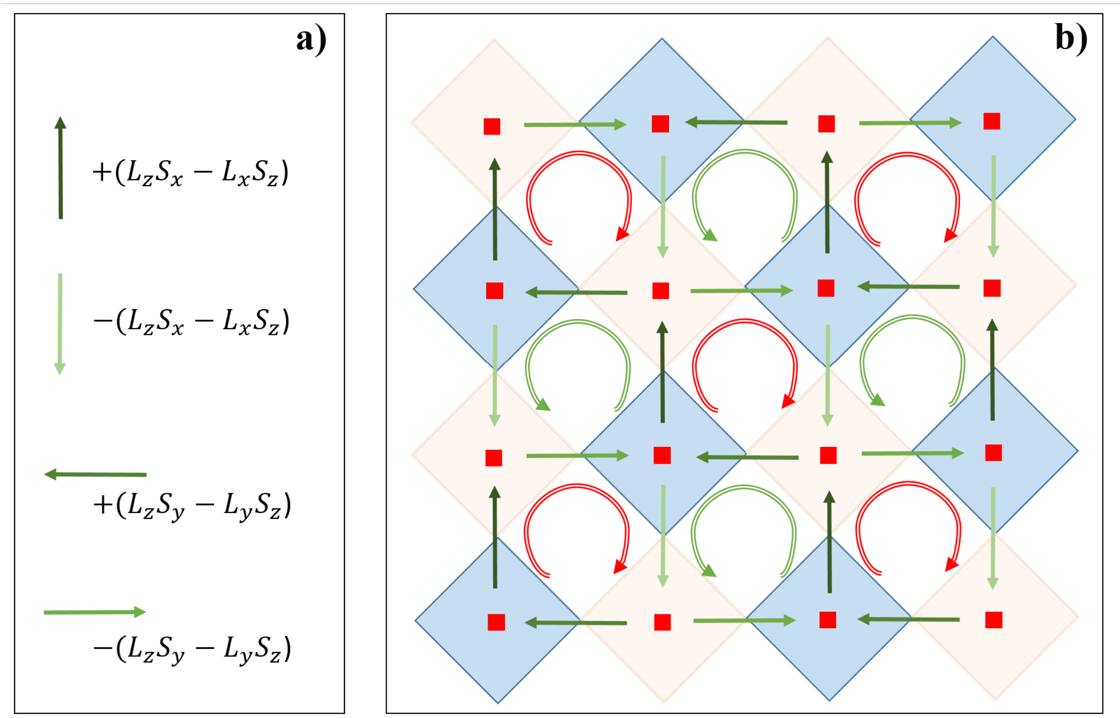

FIG. 4. **Schematic configuration of a C_4 rotational invariant spin-orbital chiral state. a** The arrows indicate the current direction on a given bond associated with the corresponding spin-orbital momentum. **b** Schematic representation of a spin-orbital chiral state with C_4 rotation symmetry.

magnetic phases with similar symmetry content with respect to mirror and time reversal symmetry and based on a long-range spatial order of Ru spin moments.

II. SPIN RESOLVED CIRCULARLY POLARIZED ARPES: MATRIX ELEMENTS AND ORBITAL ANGULAR MOMENTUM

We report here the main steps to deduce the expressions for the dichroic and spin-dichroic ARPES transition amplitudes. To this aim, we follow the derivation reported in Ref. [3] and adapt it to the spin-dichroic case too. The starting point is to consider that the circular dichroic signals probed by ARPES can be expressed through the normalized amplitude as

$$D(k) = \sum_{\sigma} \frac{[I^R_\sigma - I^L_\sigma]}{I^R_\sigma + I^L_\sigma}\,. \tag{25}$$

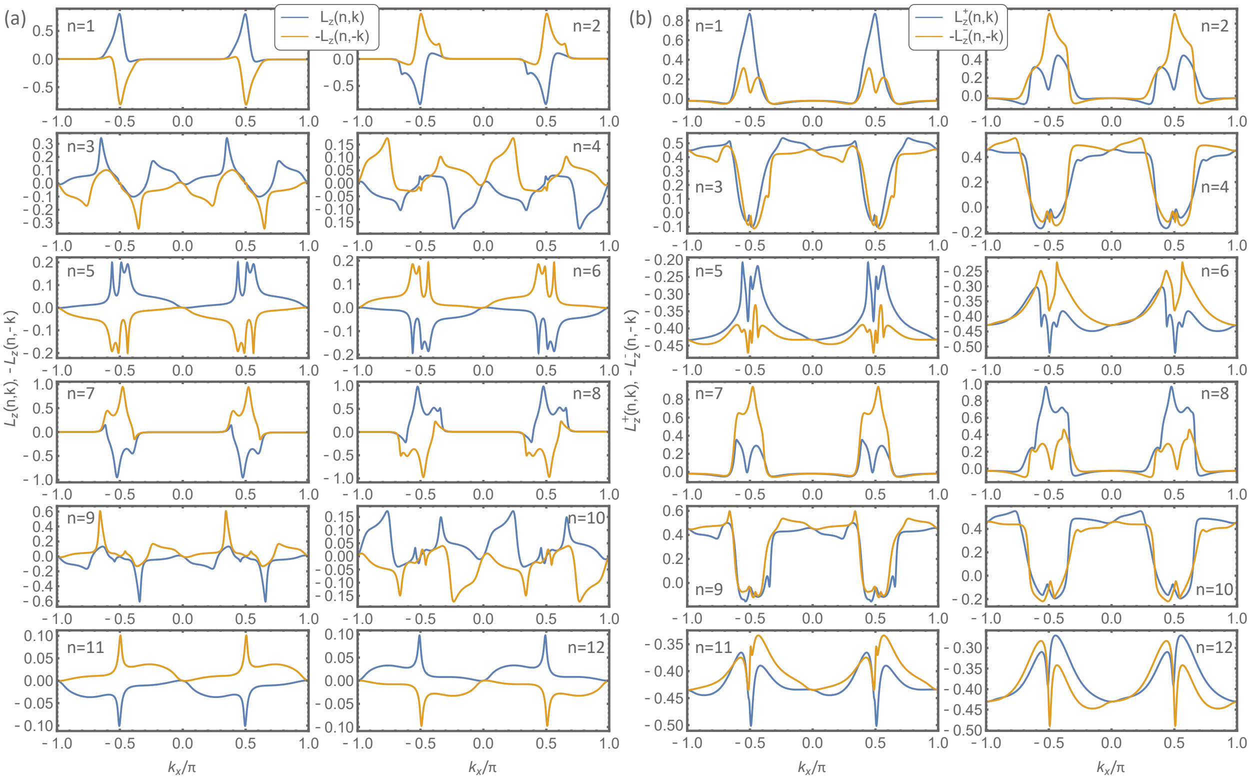

FIG. 5. **Orbital and spin-orbital textures in the presence of canted antiferromagnetism and staggered octahedral rotation.** **a** orbital moment $L_z(n,k)$ and **b** spin projected orbital orbital moment $L_z^{\pm}(n,k)$ for all bands $|\psi_{n,k}\rangle$ evaluated along the $k_y = 0$ direction. The amplitudes of the magnetizations are: $\boldsymbol{M}_{\text{stg}} = (0,0,0.1)$ and $\boldsymbol{M}_{\text{uni}} = (0.02,0,0)$ in units of eV$(g\,\mu_B)^{-1}$ (g is the electron g-factor and μ_B is the Bohr magneton.

The sum over the final state spin σ indicates the spin-integrated nature of the detection approach. Thus, one can also introduce the spin-resolved dichroic amplitude as given by

$$D_s(k) = \frac{\left[I_s^R - I_s^L\right]}{I_s^R + I_s^L}\,, \tag{26}$$

here for convenience we assume that the spin configuration is along the z-axis and are labeled by $s = +(-)$ to indicate the $\uparrow_z$ ($\downarrow_z$) spin states, respectively. Similar expressions can be derived for the all the other spin orientations.

To calculate the amplitude of the dichroic and spin-dichroic signal one has to evaluate the transition probability I for an optical excitation between the initial and final states. The interaction with the photon is given by $H_{int} = \mathbf{A}\cdot\mathbf{p}$. The vector potentials are $\mathbf{A} = \frac{\epsilon_1 + i\epsilon_2}{\sqrt{2}}$ for right circularly polarized photons, and $\mathbf{A}^* = \frac{\epsilon_1 - i\epsilon_2}{\sqrt{2}}$ for left circularly polarized photons. Hence, the vector $\mathbf{A}\times\mathbf{A}^* = -i\hat{\mathbf{k}}_{ph}$ sets out the incident photon direction. For convenience and clarity, we report the main

steps and expressions of the derivation to show the link of the ARPES intensity with the orbital and spin-projected angular momentum. Let us consider as initial state the Bloch configuration with momentum k that is given by $|\psi_k^I\rangle = \frac{1}{\sqrt{N}} \sum_{i,\alpha,\sigma} \exp[ik \cdot r_i] u_{\alpha,\sigma}(k)|i, \alpha, \sigma\rangle$, where $|u\rangle = \sum_{\alpha,\sigma} u_{\alpha,\sigma}(k)|i, \alpha, \sigma\rangle$ is the Wannier configuration centered at the site r_i associated with the t_{2g} orbitals labeled by $\alpha = xy, xz, yz$ and spin σ. A plane-wave state is assumed for the final state $|F\rangle = \int \exp[ik_F r]|r\rangle$ [6, 7] with k_F being the Fermi wave vector. We recall that the expected value of the orbital angular momentum evaluated on the state $|\psi_k^I\rangle$ is given by $\langle \hat{L} \rangle = i \sum_s u_s \times u_s^*(k)$, with $\mathbf{u}_s = (u_{yz,s}, u_{zx,s}, u_{xy,s})$ and the spin projected orbital components are expressed as $\langle \hat{L}(1 + s\hat{\sigma}_z)\rangle = iu_s(k) \times u_s^*(k)$, with $s = \pm$ singling out the spin up and down configuration with respect to the z-direction, respectively. Following the derivation in Ref. [3], one can show that the spin resolved transition probability can be generally expressed as

$$D_\sigma = \frac{(\mathbf{A} \times \mathbf{A}^*) \cdot \nabla g_\sigma \times \nabla g_\sigma^*}{[(\mathbf{A} \cdot \nabla g_\sigma)(\mathbf{A}^* \cdot \nabla g_\sigma^*) + (\mathbf{A}^* \cdot \nabla g_\sigma)(\mathbf{A} \cdot \nabla g_\sigma^*)]} \tag{27}$$

with

$$g_\sigma(k_F) = \mathbf{u}_\sigma \cdot \nabla_{k_F} f(k_F) \tag{28}$$

where the gradients are respect to the Fermi wave vector k_F, and $f(k)$ is the Fourier transform of the part of the Wannier function that depends only on the radial distance $r - r_i$ related to the atomic center at r_i. The denominator of D is always positive and has a minor role in the dependence of the matrix elements from the orbital angular momentum. The key quantity for our purposes is given by the factor $\Gamma_s = \nabla g_s \times \nabla g_s^*$. One can show [3] that the term Γ_s can be expressed as

$$\Gamma_s = \frac{1}{2} \varepsilon^{\alpha\beta\gamma} (\mathbf{u}_s \times \mathbf{u}_s^*)_\alpha \nabla P_\beta f \times \nabla P_\gamma f \tag{29}$$

$$= i\frac{1}{2} \varepsilon^{\alpha\beta\gamma} \langle \hat{L}_\alpha(1 + s\hat{\sigma}_z)\rangle \nabla P_\beta f \times \nabla P_\gamma f \tag{30}$$

with the vector $\mathbf{P}$ having the following components (Q_{yz}, Q_{zx}, Q_{xy}) with $Q_{ij} = \partial_i \partial_j$. We notice that while the structure of the orbital angular momentum is similar to that of p-orbitals because we are using an effective $L = 1$ manifold for the t_{2g} sector, the second order differential operator Q_{ij} takes into account the different structure of the orbital configurations. In a similar way, one can show that the spin integrated Γ amplitude is given by

$$\Gamma = i\frac{1}{2} \varepsilon^{\alpha\beta\gamma} \langle \hat{L}_\alpha \rangle \nabla P_\beta f \times \nabla P_\gamma f \,, \tag{31}$$

with $\varepsilon^{\alpha\beta\gamma}$ the Levi-Civita tensor. Then, one can see that the amplitude Γ and thus the dichroic signal is proportional to the projected orbital angular momentum components $\langle \hat{L}_\alpha \rangle$ with respect

to the incident photon direction. Additionally, the spin-dichroic signal is proportional to the spin-projected orbital angular momentum as given $\langle \hat{L}_\alpha(1+s\hat{\sigma}_z)\rangle$ with $s = \pm$ for projecting spin up and down configurations, respectively. The remaining form factors depend on the Fermi momentum and on the photon energy. Since our study aims to have a qualitative understanding of the anomalies in the transition amplitude probed by Spin CD-ARPES, their contribution does not affect the conclusions of our findings.

In the employed experimental setup the spin orientation is selected in a direction that is perpendicular to the surface (i.e. z) and the incident photon direction is primarily selecting the out-of-plane z-component of the orbital angular momentum. Hence, the dichroic and spin-dichroic signal are proportional to $\langle \hat{L}_z\rangle$ and $\langle \hat{L}_z(1 \pm \hat{\sigma}_z)\rangle$ as considered in the manuscript.

III. CURRENT DRIVEN PHASE BY COULOMB INTERACTION

Let us now present how the electronic current phase arises as a broken symmetry state with a nonvanishing expectation value of the current operator on the Ru-Ru bond due to the Coulomb interaction. Indeed, in order to demonstrate this point one needs to introduce the spin-orbital asymmetric operator

$$\phi^{\alpha\beta}_{\sigma,\sigma'}(l,m) = i\left(c^\dagger_{\alpha,\sigma}(l)c_{\beta,\sigma'}(m) - c^\dagger_{\beta,\sigma'}(m)c_{\alpha,\sigma}(l)\right) \quad (32)$$

for the $l-m$ bond between two Ru atoms with position identified by the coordinates R_l and R_m. Here, $c_{\alpha,\sigma}(l)(c^\dagger_{\alpha,\sigma}(l))$ are the annihilation (creation) operators associated with an electronic state with α orbital and spin σ at the atomic site R_l. Then, the spin and orbital dependent terms that build up the density-density inter-site Coulomb interaction U_{lm} for a generic $l-m$ bond can be written in the following form

$$U_{lm}n_{\alpha,\sigma}(l)n_{\beta,\sigma'}(m) = -\frac{1}{2}U_{lm}(\phi^{\alpha\beta}_{\sigma,\sigma'}(l,m))^\dagger\phi^{\alpha\beta}_{\sigma,\sigma'}(l,m) + \frac{1}{2}U_{lm}\left(n_{\alpha,\sigma}(l) + n_{\beta,\sigma'}(m)\right) \quad (33)$$

where the orbital and spin resolved density operators, $n_{\alpha,\sigma}(l)$, are defined as $n_{\alpha,\sigma}(l) = c^\dagger_{\alpha,\sigma}(l)c_{\alpha,\sigma}(l)$. Hence, by decoupling the quartic term, one can introduce an order parameter associated with the expectation value of $\phi^{\alpha\beta}_{\sigma,\sigma'}(l,m)$ and express the interaction as

$$U_{lm}n_{\alpha,\sigma}(l)n_{\beta,\sigma'}(m) \sim -\frac{1}{2}\left[\langle\phi^{\alpha\beta}_{\sigma,\sigma'}(l,m)\rangle(\phi^{\alpha\beta}_{\sigma,\sigma'}(l,m))^\dagger + h.c. - |\langle\phi^{\alpha\beta}_{\sigma,\sigma'}(l,m)\rangle|^2\right] \quad (34)$$

where the average value indicates the summation over all the electronic states weighted by the Fermi distribution function. Taking into account the spin-orbital order parameters on the $l-m$

bond, by suitable superposition of the ϕ operators one can construct a bond current order parameter that is given by the expectation value of the following orbital and spin-orbital quadrupole current operators:

$$J^{o}_{i,j}(l,m) = i\left(\vec{c}^{\,\dagger}(l)\hat{L}_i\hat{L}_j\vec{c}(m) - h.c.\right)$$
$$J^{so}_{i,j}(l,m) = i\left(\vec{c}^{\,\dagger}(l)\hat{L}_i\hat{S}_j\vec{c}(m) - h.c.\right) \quad (35)$$

with $\vec{c}^{\,\dagger}(l) = \left[c^{\dagger}_{xy,\uparrow}(l), c^{\dagger}_{yz,\uparrow}(l), c^{\dagger}_{zx,\uparrow}(l), c^{\dagger}_{xy,\downarrow}(l), c^{\dagger}_{yz,\downarrow}(l), c^{\dagger}_{zx,\downarrow}(l)\right]$ and $i, j \in \{x, y, z\}$.

---

[1] V. Zabolotnyy, D. Evtushinsky, A. Kordyuk, T. Kim, E. Carleschi, B. Doyle, R. Fittipaldi, M. Cuoco, A. Vecchione, and S. Borisenko, Renormalized band structure of Sr_2RuO_4: A quasiparticle tight-binding approach, Journal of Electron Spectroscopy and Related Phenomena **191**, 48 (2013).

[2] S. R. Park, C. H. Kim, J. Yu, J. H. Han, and C. Kim, Orbital-angular-momentum based origin of rashba-type surface band splitting, Phys. Rev. Lett. **107**, 156803 (2011).

[3] J.-H. Park, C. H. Kim, J.-W. Rhim, and J. H. Han, Orbital rashba effect and its detection by circular dichroism angle-resolved photoemission spectroscopy, Phys. Rev. B **85**, 195401 (2012).

[4] B. Kim, P. Kim, W. Jung, Y. Kim, Y. Koh, W. Kyung, J. Park, M. Matsunami, S.-i. Kimura, J. S. Kim, J. H. Han, and C. Kim, Microscopic mechanism for asymmetric charge distribution in rashba-type surface states and the origin of the energy splitting scale, Phys. Rev. B **88**, 205408 (2013).

[5] M. T. Mercaldo, P. Solinas, F. Giazotto, and M. Cuoco, Electrically tunable superconductivity through surface orbital polarization, Phys. Rev. Appl. **14**, 034041 (2020).

[6] A. Damascelli, Z. Hussain, and Z.-X. Shen, Angle-resolved photoemission studies of the cuprate superconductors, Rev. Mod. Phys. **75**, 473 (2003).

[7] S. Moser, An experimentalist's guide to the matrix element in angle resolved photoemission, Journal of Electron Spectroscopy and Related Phenomena **214**, 29 (2017).
